# Effects of leachates from UV-weathered microplastic on the microalgae *Scenedesmus vacuolatus*

**DOI:** 10.1007/s00216-021-03798-3

**Published:** 2021-12-22

**Authors:** Christoph D. Rummel, Hannah Schäfer, Annika Jahnke, Hans Peter H. Arp, Mechthild Schmitt-Jansen

**Affiliations:** 1 Department of Bioanalytical Ecotoxicology, Helmholtz Centre for Environmental Research – UFZ, Permoserstr. 15, 04318 Leipzig, Germany; 2 Department of Ecological Chemistry, Helmholtz Centre for Environmental Research – UFZ, Permoserstr. 15, 04318 Leipzig, Germany; 3 Institute for Environmental Research, RWTH Aachen University, Worringerweg 1, 52074 Aachen, Germany; 4 Norwegian Geotechnical Institute (NGI), Ullevål Stadion, P.O. Box 3930, 0806 Oslo, Norway; 5 Department of Chemistry, Norwegian University of Science and Technology (NTNU), 7491 Trondheim, Norway

**Keywords:** Microplastic, Leachates, Microalgae, Artificial weathering, Mode of toxic action, Electronic waste

## Abstract

Plastics undergo successive fragmentation and chemical leaching steps in the environment due to weathering processes such as photo-oxidation. Here, we report the effects of leachates from UV-irradiated microplastics towards the chlorophyte *Scenedesmus vacuolatus*. The microplastics tested were derived from an additive-containing electronic waste (EW) and a computer keyboard (KB) as well as commercial virgin polymers with low additive content, including polyethylene (PE), polyethylene terephthalate (PET), polypropylene (PP), and polystyrene (PS). Whereas leachates from additive-containing EW and KB induced severe effects, the leachates from virgin PET, PP, and PS did not show substantial adverse effects in our autotrophic test system. Leachates from PE reduced algae biomass, cell growth, and photosynthetic activity. Experimental data were consistent with predicted effect concentrations based on the ionization-corrected liposome/water distribution ratios (*D*_lip/w_) of polymer degradation products of PE (mono- and dicarboxylic acids), indicating that leachates from weathering PE were mainly baseline toxic. This study provides insight into algae toxicity elicited by leachates from UV-weathered microplastics of different origin, complementing the current particle- vs. chemical-focused research towards the toxicity of plastics and their leachates.

**Graphical abstract:**

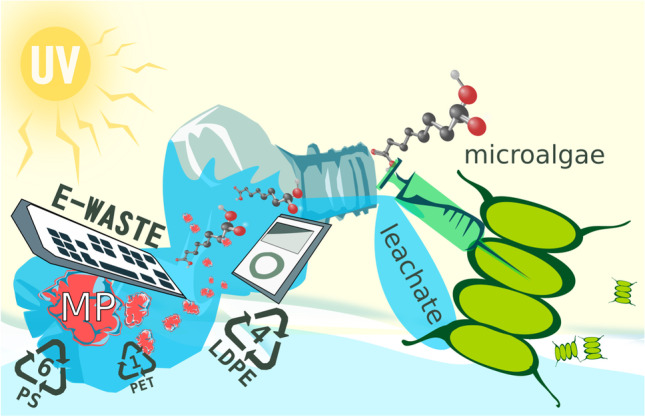

**Supplementary Information:**

The online version contains supplementary material available at 10.1007/s00216-021-03798-3.

## Introduction

Interactions between plastic debris and biota have been reported for diverse taxa at different trophic levels [1]. In most ecotoxicological studies, this interaction focuses on effects from the ingestion or uptake of particles into the test organisms. As globally distributed primary producers, microalgae have a high potential to interact with microplastics [2]. Adverse effects of microplastics towards microalgae may either stem directly from the physical presence of particles or by the secondary effects from leaching chemicals [3, 4]; however, these are difficult to distinguish due to their frequent co-occurrence. The first process may lead to particle adsorption, aggregation, loss of membrane integrity, or shading effects as discussed in previous studies [2, 5, 6]. In contrast, the second process of indirect effects induced by chemicals leaching from microplastics has gained little attention for microalgae so far, despite many algae being able to colonize on plastic surfaces [7, 8]. The few studies that have considered leachates have confirmed the expected adverse effects by additive leaching [9, 10]. The presence, release, and adverse effects of common plastic additives has been reported for a variety of polymer materials [11–13], yet it remains unclear whether toxicity of leachates is driven by additives, polymer residuals such as mono- or oligomers, or degradation products after weathering.

The electronic waste microplastic (EW) herein was derived from an e-waste recycling/sorting facility and was previously reported to contain flame retardants, bisphenol A, polychlorinated biphenyls and antimony [14–16, 19]. Also included was microplastic generated from a key board (KB), which was also expected to contain chemical mixtures of additives, based on detectable effects of KB leachates in reporter gene assays [17]. Effects of leachate waters of these two sample types were compared to the leachate effects of comparatively additive-free pre-production polymer pellets that mainly contained polymer residuals and degradation products of polymers as a result of artificial weathering. Our previous research demonstrated that effects of plastic leachates on a cellular level may be induced by plastic degradation products solely, i.e., largely excluding effects of intentionally added plastic additives that may leach from the test material [17]. In Rummel et al. [17], we described the induction of cellular stress responses such as oxidative stress by plastic leachates from raw polymer pellets. Furthermore, adverse effects were elevated when leaching was conducted under strong UV light treatment that caused photo-oxidation of the polymers [17]. To further investigate the ecotoxicological relevance of these plastic leachates, in vivo, in this study, we test the potency of these leachates to induce adverse effects towards ecologically important low-trophic level representatives: microalgae. Following the OECD No. 201 test guideline [18], we addressed potential impairment of algal growth and a key physiological process, namely photosynthesis, in one test assay. Microalgae were chosen as suitable test organisms since we could ensure the test accuracy of cell-based tools but with increased biological complexity in comparison to our previously used test systems [17] by using a population-based test. Furthermore, the test system offers options to assess specific effects like the inhibition of photosynthesis and therefore to address a specific potential mode of action (MoA) of toxicants. Microalgae are representative primary producers that provide important ecosystem functions.

In this study, we hypothesize that plastic leachates from accelerated photo-induced polymer breakdown products generated in artificial seawater cause impaired photosynthetic activity and cell growth in microalgae. To test this hypothesis, we investigated the toxicity of leachate waters from microplastics with large and trace amount additives. The microplastics with large amounts of additives were derived from consumer products, namely electronic waste (EW) and a keyboard plastic (KB). The EW investigated herein was derived from an e-waste recycling/sorting facility and was previously reported to contain flame retardants, antimony, bisphenol A, and polychlorinated biphenyls [14–16, 19]. As an electronic device, the KB was also expected to contain chemical mixtures of additives, based on detectable effects of KB leachates in reporter gene assays [17]. The microplastic with trace/negligible amounts of additives was derived from pre-production polymer pellets of polyethylene (PE), polyethylene terephthalate (PET), polypropylene (PP), and polystyrene (PS), which mainly contained polymer residuals and degradation products of polymers because of artificial UV-weathering treatment to induce photo-oxidation.

To obtain further insights into the MoA of leachates from UV-weathered microplastic, we investigated whether polymer degradation products (carboxylic acids) previously identified as PE degradation products by Gewert et al. [20] have the potential to explain the observed algae toxicity. The observed biological responses were discussed in relation to estimated algae effect concentrations that elicit 50% of the maximum effect (EC_50_ values) for baseline toxicity for these carboxylic acids as well as other additives for the EW by quantitative structure relationships by Altenburger et al. [21]. Finally, the data were compared to effect data derived from cell-based bioassays from Rummel et al. [17].

## Material and methods

### Test materials and chemicals

The test polymers PE, PET, PS, and PP were purchased from Goodfellow (Hamburg, Germany). Diuron (as positive control, CAS: 330–54-1, Sigma-Aldrich, Steinheim, Germany), methanol (MeOH), and ethylacetate (EtOAc) (HPLC-grade, ≥ 99.9%, Honeywell Riedel de Haen, Fisher Scientific GmbH, Schwerte, Germany), sodium hydrogen carbonate (CAS 144–55-8, Sigma-Aldrich, Steinheim, Germany), ingredients for the Grimme Boardman (GB) medium [22], and LC-grade water were purchased from Optima™ Fisher Chemical (Reinbach, Suisse). Oasis HLB Plus (Waters GmbH, Eschborn, Germany) with 225 mg sorbent were used for solid-phase extraction (SPE) of the chemicals that leached from the test materials during artificial weathering. Mono- and dicarboxylic acids (α,ω position) of carbon chain lengths of C5, C7 − C12, C14, C16, and C18 were purchased from Sigma-Aldrich (Steinheim, Germany) (for detailed information see Table S1 of the Electronic Supplementary Material (ESM)).

### Leachates

For each polymer type, EW, KB, PE, PET, PP, and PS, 200 mL of aqueous leachates were generated in triplicates as described in Rummel et al. [17] and in section S1 Test Polymers (ESM). Briefly, 50 g of milled polymer material < 300 µm was artificially weathered in artificial seawater (ASW, Instant Ocean® (Blacksburg, VA, USA at 35 g/L) using a strong UV light source (OSRAM Supratec HTC400-241 R7s UVA/UVB lamp) in a rotating wheel to guarantee constant mixing (hereafter named “UV” samples). Dark controls were leached in the same manner but without UV light exposure (hereafter named “DC”). To account for any background contamination, three procedural blanks were generated following the entire leachate preparation protocol but without any polymer. After the weathering treatment, microplastic particles were filtrated to yield the 200 mL of leachate water only, which was then enriched via solid-phase extraction (SPE, HLB Plus Oasis cartridges, 225 mg, Waters GmbH, Eschborn, Germany). Additionally, four SPE blanks generated by enriching 200 mL LC-grade water were tested for potential background effects by the SPE enrichment. After enrichment, the SPE cartridges were rinsed with 10 mL of LC-grade water to eliminate salt residues in the sorbents. After drying for 2 h under vacuum in the manifold, the SPE cartridges were stored at room temperature in the dark. Following elution with EtOAc and MeOH and solvent evaporation, the samples were re-dissolved in 1 mL of MeOH for testing. This procedure resulted in an enrichment factor (EF) of 200 based on the aqueous leachate (200 mL) and final methanolic sample volume (1 mL) (for details, see Supporting Information in Rummel et al. [17]).

### Exposure

To study the effects of microplastic leachates towards microalgae, a miniaturized high-throughput algae assay based on the OECD guideline No. 201 (Freshwater Alga Growth Inhibition Test, OECD [18]) was used. In this test setup, a synchronized culture of the unicellular green algae *Scenedesmus vacuolatus,* cultivated in GB medium, was exposed to the test material leachates in a 96-well plate (see Figure S2 A, ESM). Aliquots of the leachates were blown down to dryness, re-dissolved in GB medium, and diluted serially 1:2 in the well plate with 10 dilution steps. Concentrations in the bioassays of enriched leachates are given as the product of the SPE enrichment factor of 200 and the dilution factor from the serial dilution resulting in the relative enrichment factor (REF). The highest tested relative enrichment factor (REF) was REF = 197.8. Negative controls contained GB medium only. For positive controls, reference cells were exposed to the photosystem II inhibitor Diuron [23] at 1.17 µmol/L as the highest test concentration, eliciting 100% effect. After verifying the absence of any interfering autofluorescence of the samples using a microplate fluorescence reader (Spectra Max Gemini EM, Molecular Devices, San Jose, USA), 15 µL of algae suspension were added to each well with a final algae density of 7.5 × 10^4^ cells/mL with a total volume of 150 µL per well. Plates were sealed with Parafilm and LED day light–exposed for 24 h at 300 rpm rotation and 28 °C in a Multitron incubator (Infors, Bottmingen, Germany).

Chlorophyll *a* autofluorescence, as a representative biological effect parameter for biomass, was measured 2 h after the addition of algae and after 24 h of exposure. Cell density, indicating cell growth and/or growth inhibition, was determined using a FACSCelesta (BD Biosciences, NJ, USA) instrument after 24 h of exposure. Furthermore, we determined the photosynthetic capacity as maximum quantum yield (Yield I (YI)) and effective quantum yield (Yield II (YII)) after 2 h and 24 h of exposure using the Imaging PAM Chlorophyll Fluorometer (M-series, Heinz Walz GmbH, Effeltrich, Germany) (see Figure S2 B, ESM).

### Data analyses

Algae growth rates were calculated based on the Chlorophyll *a* autofluorescence and details are described in section S2 Data Analyses (ESM). For the other parameters, cell densities and the photosynthetic yields YI and YII after 2 h and 24 h of exposure, the measured values were used for the calculation of the relative inhibition to controls (in percent (%)) without background subtraction (section S2 Data Analyses, ESM).

Analogously to Rummel et al. [17], effect units (EU) for the parameter autofluorescence (EU_fluo_) or cell density (EU_cell_) were calculated as the inverse EC_50_ value (i.e., in the unit 1/REF). If all three tested replicates of a sample type resulted in a measurable effect, the mean and the 95% confidence interval were calculated for the triplicates to facilitate comparison between samples.

Gewert et al. [20] confirmed the presence of carboxylic acids in aqueous leachates from the identical UV-weathered virgin test polymers, for which we observed effects in reporter gene assays in our previous work [17]. Hence, we tested a set of mono- and dicarboxylic acids (α,ω position) of different carbon chain lengths of C5, C7 − C12, C14, C16, and C18 in the microalgae bioassay to investigate their potential to cause algae toxicity.

We applied a quantitative structure–activity relationship QSAR for the applied algae test system [21] to predict EC_50_ values of baseline toxicity of the carboxylic acids towards microalgae (Eq. 1). This QSAR is based on the hydrophobicity (*K*_ow_) of the chemical of investigation. However, at the applied pH of 7.0 in the assay, the carboxylic acids will fully dissociate and be present in their anionic form, for which the cellular uptake is slower and lower [24]. To account for speciation, the partition coefficient between liposomes and water, *K*_lipw_, of the neutral species of the carboxylic acids was predicted by the log*K*_ow_-based QSAR by Endo et al. [25] (Eq. 2). The fraction of the neutral species was calculated using the Henderson-Hasselbalch equation from the acidity constant p*K*_a_ (taken from PubChem, predicted using SPARC or assumed at p*K*_a_ = 4.9, see Table S2 section S3 QSAR Data). Baseline toxicity values of the neutral fraction using the ionization-corrected liposome/water distribution ratios [*D*_lip/w_ (pH 7.0)] (Eq. 3) were calculated following Escher et al. [26] (Eq. 4), parameterized with the converted slope and intercept of the QSAR by Altenburger et al. [21] (Eq. 1) by replacing log*K*_ow_ by log*K*_lipw_ of Endo et al. [25] (Eq. 2) (see details in section S3, Table S2, ESM).

1 $$\log{EC}_{50}\left(\frac{mol}L\right)=-0.863\ast logK_{ow}-0.897\lbrack21\rbrack$$

2 $$\log K_{lipw}\left(neutral\right)=1.01\ast\log K_{ow}-0.12\lbrack25\rbrack$$

3 $${\log D}_{lipw}\left(pH7.0\right)=f_{neutral}\ast\log K_{lipw}+f_{ionized}\ast\left(\log K_{lipw}-1\right)\lbrack26\rbrack$$

4 $$\log{(1/EC}_{50,basline})\left(\frac{mol}L\right)=-0.855\ast{\log D}_{lipw}\left(pH7.0\right)-1.02$$

As an effect diagnostic tool, we calculated the toxic ratios (TRs, Eq. 5) between the predicted baseline toxicity (Eq. 4) and the measured effect data of the parameters fluorescence and cell density to differentiate baseline toxicity from potential specific MoAs of microplastic degradation products towards the photosystem (here: mono- and dicarboxylic acids). We consider a specific MoA to occur when the TR ranges > 10 [26]. To further confirm non-specific effects of degradation products of the polymers, the cytotoxicity values of the bioassay from our previous work published in Rummel et al. [17] (inverse IC_10_ as TU_bio_) were correlated to the apical parameters fluorescence and cell density of algae growth to further investigate baseline toxicity. To enable comparison to this former study [17], EC_10_ values for microalgae were calculated from the EC_50_ values based on Eq. 6.

5 $$TR= \frac{{predicted EC}_{50 (baseline)}}{{measured EC}_{50}}$$

6 $${EC}_{x}={\left(\frac{x}{100 - x}\right)}^{{~}^{1}\!\left/ \!\!{~}_{h}\right.}*{EC}_{50}$$

*x* = *x* % effect (here *x* = 10)

*h* = Hill slope

To estimate the percent effect explained by chemicals previously determined in the EW material, we calculated the potential mass concentrations in the leachates for several polychlorinated biphenyls (PCBs), polybrominated diphenyl ethers (PBDEs), and bisphenol A (BPA) by mass balancing (section S4 Iceberg Modelling, eq.S3–eq.S6). Applying the above-mentioned QSAR for baseline algae toxicity [21], we estimated the respective EC_50_ concentrations and effect contributions by each chemical (*i*) and summed them up in a so-called iceberg model based on the concentration addition model to estimate the effect units elicited by the known chemicals (EU_chem_) [27, 28]. Comparing EU_chem_ to EU_fluo_ or EU_cell_ gives a rough estimate of the effect contribution of the known leachate mixture to the observed total biological responses (for details, see section S4 Iceberg Modelling, Table S3, Table S4, ESM).

## Results

### Effects of microplastic leachates

**Fig. 1:**
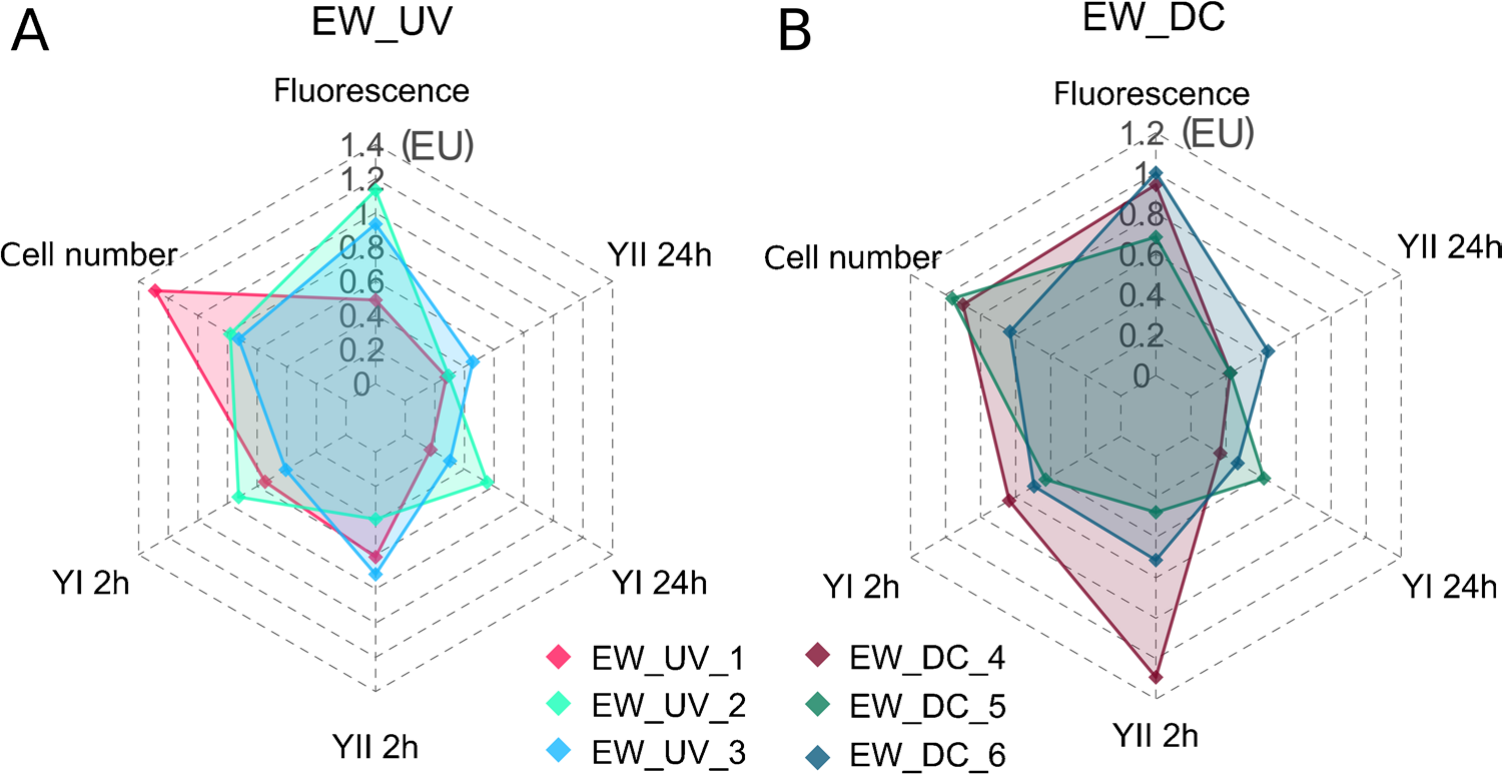
Radar plots of the effect unit values (EU = inverse EC_50_ values) of the different effect parameters of leachates from the EW microplastics following UV light (UV, in triplicates UV_1-3, **A**) and dark control (in triplicates DC_4-6, **B**) treatments tested in microalgae. The effect parameters presented are fluorescence, cell densities, and photosynthetic yield I and II after 2 h or 24 h (YI2h, YII2h, YI24h, and YII24h) of exposure, respectively. The EU scale corresponds to the inverse relative enrichment factor (REF) [unit *L*_bioassy_/*L*_water_]

When testing microplastic leachates from different polymer types in microalgae, the growth-based apical parameters (autofluorescence and cell densities) were more responsive than photosynthesis inhibition (YI and YII) (Fig. 1A, B, Table S5, ESM). Therefore, these two apical parameters were chosen for subsequent comparisons between samples.

**Fig. 2:**
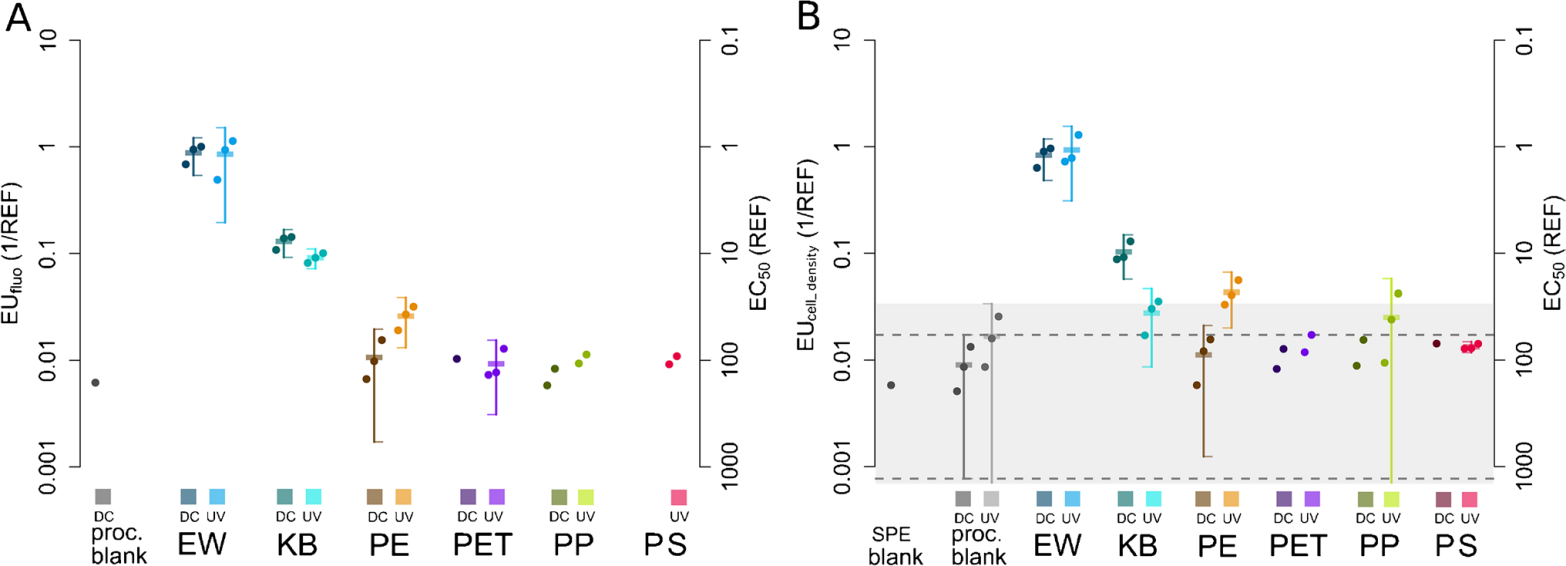
Effect units (EU, 1/REF [*L*_bioassay_/*L*_water_]) of Chl *a* autofluorescence (**A**) and cell density (**B**) inhibition of microalgae *S. vacuolatus* exposed to leachates from microplastics containing additives (EW, KB) and largely additive-free polymers (PE, PET, PP, and PS). EUs (left axis) are given as the inverse EC_50_ to facilitate association of high toxicity with high values; EC_50_ is additionally given as the scale to the right (unit REF [*L*_water_/*L*_bioassay_]). Leachates were generated under UV light (UV) and dark control conditions (DC) in triplicates (single data points). If all triplicates caused measurable effects, the mean and 95% confidence interval were calculated and depicted as boxes and whiskers. Comparison to procedural blanks is facilitated by the dotted dashed line and grey shaded area in **B** (not in **A** since none of the different triplicate blanks elicited effects on Chlorophyll *a* fluorescence). Missing values indicate the absence of measurable effects at the tested concentrations REF < 198

Only the EW and KB induced effects on all measured effect parameters whereas effects of the test polymer leachates could only be observed on algal growth based on Chlorophyll *a* autofluorescence indicative for biomass, as well as cell densities indicative for cell growth inhibition (Fig. 1, Fig. 2, Table S5, ESM). For EW and KB, the photosynthetic capacity was less affected after 24 h after dosing (YI and YII 24 h) compared to the 2 h measurements (YI and II 2 h, Fig. 1A and B). Only one of the three dark control (DC) procedural blanks negatively affected the fluorescence of *S. vacuolatus* (Fig. 2A). No effects on the fluorescence could be detected for the SPE and the UV light–treated blanks. The EW caused a decrease in the Chlorophyll *a* fluorescence compared to unexposed microalgae at mean EU values and standard deviations of EW_DC_ = 0.88 ± 0.17 and EW_UV_ = 0.85 ± 0.32. Interestingly, the UV light–irradiated PE_UV_ showed higher adverse effects on microalgae compared to its dark control PE_DC_ for the effect parameters fluorescence and cell densities (Fig. 2A, B). For the remaining polymers PET, PP, and PS, measurable effects were not reproduced in each replicate (*n* = 3).

One SPE blank and all replicates of the procedural blanks (DC and UV) resulted in detectable effects on the algae cell densities (Fig. 2B, Table S5, ESM). Whereas EW showed similarly high EU mean values for DC and UV treatments of EW_DC_ = 0.83 ± 0.18 and EW_UV_ = 0.93 ± 0.3, KB_UV_ displayed a much lower effect potency than KB_DC_. (KB_DC_ = 0.10 ± 0.02, KB_UV_ = 0.03 ± 0.01). As observed for Chlorophyll *a* fluorescence inhibition, PE_UV_ induced four times higher effects towards algae cell densities than PE_DC_. Noteworthy, most of the observed effects on cell densities for the tested additive-free polymer leachates were in the range of the corresponding procedural blanks except PE_UV_ which differed from the corresponding blanks.

The EW elicited the most explicit effects, and the effects on Chlorophyll *a* fluorescence and cell densities exhibited the highest EU_bio_ values (EC_fluo_ (EW_DC or UV_) = 0.49–1.1; EC_cell_ (EW_DC or UV_) = 0.63–1.29) in contrast to the photosynthetic yield (YI/II 2–24 h (EW_DC_ or EW_UV_) = 0.17–1.09) (Fig. 2, Table S5, ESM). Photosynthesis was adversely affected mainly by the leachates from EW and KB as well as by the PE leachates (DC and UV) (Table S5, ESM). PE_UV_ generally showed stronger adverse effects to YI and YII compared to PE_DC_ and was within the range of the effects observed for EW and KB leachates. Furthermore, EC_50_ increased after 24 h of exposure compared to 2 h after dosing (Table S5, ESM). PET, PP, and PS showed minor effects towards microalgae and, if detectable, they were mostly within the range of the blanks (Fig. 2, Table S5, ESM).

### Effects elicited by polymer degradation products

**Fig. 3:**
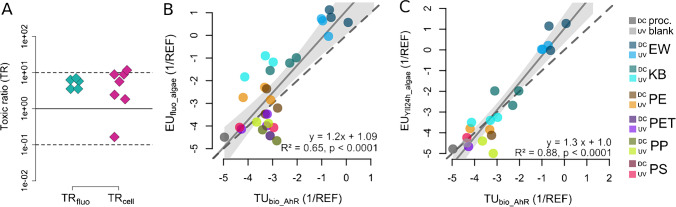
**A** Toxic ratios (TRs) for mono- and dicarboxylic acids (C5–C18) tested in the microalgae test with measured effect parameters for autofluorescence (fluo) and cell densities (cell). **B** Statistically significant correlations between cell-based assay data [17] and microalgae data (this study) were observed and confirmed by the linear regression of the apical observation parameter fluorescence for microalgae (fluo_algae) as a function of toxic units (TU_bio_) of the specific effects in the AhR assay (regression parameters, coefficient of determination, and *p*-value are included in the plot, log-transformed data). **C** Statistically significant correlation of TU_bio_ in the AhR assay and the photosynthetic activity at 24 h after dosing for the microalgae (EU_YII24h_algae_, log-transformed). Dashed lines in B and C represent the 1:1 line which corresponds to a 1:1 correlation. Color codes for B and C show the different polymer types with respective dark controls (DC) and UV-irradiated (UV) weathering treatments

When testing mono- and dicarboxylic acids in the microalgae test at the highest soluble concentrations, mainly the monocarboxylic acids resulted in detectable effects (Table 1). Only dicarboxylic acids of carbon chain lengths C5 and C7 resulted in measurable effects whereas mono- and dicarboxylic acids of carbon chain lengths greater than eleven did not generally cause algae toxicity or impairment of the photosystem. Most of the calculated TRs for the growth-based effect parameters (fluorescence and cell densities) were located in a narrow range of 1 < TRs < 10 except for two carboxylic acids that were outliers above and below these thresholds (Fig. 3A).

**Table 1:** QSAR and effect data of mono- and dicarboxylic acids tested in the microalgae test system

Substance	CAS	MW	log *P*_ow_ [L_w_/L_o_]	QSAR EC_50_ [mM]^d^	EC_50_ fluorescence [mM]	EC_50_ cell density [mM]
Pentanoic acid	109–52-4	102.13	1.39^a^	51.59	14.49	9.18
Pentanedioic acid	110–94-1	132.12	0.256 ^a^	1561.30	268.59	174.54
Heptanoic acid	111–14-8	130.19	2.42 ^a^	6.99	1.11	0.60
Heptanedioic acid	111–16-0	160.17	0.61 ^a^	258.82	ND	140.97
Octanoic acid	124–07-2	144.2	3.05 ^a^	1.74	0.49	0.19
Octanedioic acid	505–48-6	174.20	1.21^b^	112.91	ND	ND
Nonanoic acid	112–05-0	158.24	3.4 ^a^	0.94	1.35	0.39
Nonanedioic acid	123–99-9	188.22	1.57 ^a^	35.27	ND	ND
Decanoic acid	334–48-5	172.27	4.09 ^a^	0.24	ND	1.45
Decanedioic acid	111–20-6	202.25	2.19 ^b^	28.10	ND	ND
Undecanoic acid	112–37-8	186.30	4.42 ^a^	0.13	ND	ND
Undecanedioic acid	1852–04-6	216.28	2.8 ^c^	6.99	ND	ND
Dodecanoic acid	143–07-7	200.32	4.6 ^a^	0.08	ND	ND
Dodecanedioic acid	693–23-2	230.30	3.17 ^b^	3.16	ND	ND
Tetradecanoic acid	544–63-8	228.38	6.1 ^a^	0.005	ND	ND
Tetradecanedioic acid	821–38-5	258.36	4.3 ^b^	2.12	ND	ND
Hexadecanoic acid	57–10-3	256.43	7.2 ^a^	5.10E-04	ND	ND
Octadecanoic acid	57–11-4	284.48	8.23 ^a^	6.99E-05	ND	ND

^a^Experimental (source: PhysPropNCCT)

^b^Computed by XLogP3 3.0 (PubChem release 2019.06.18)

^c^Safety data sheet

^d^Calculated by quantitative structure relation by Altenburger et al. [21]

*ND* not detected

### Effect diagnostics

We correlated the results for cytotoxicity obtained using reporter gene bioassays from Rummel et al. [17] with the microalgae test results (correlation to cytotoxicity of the aryl hydrocarbon receptor assay AhR CALUX [29, 30] shown in Fig. 3B, C). Here, calculating the EC_10_ values for the microalgae test results was necessary to facilitate a comparison to the EC_10_ and the inverse effect units (EU_bio_) and toxic units (TU_bio_) derived in the bioassay. Linear regressions of the microalgae test data as a function of reporter gene cytotoxicity (TU_bio_) resulted in statistically significant correlations in many cases with slopes close to one (Fig. 3B, C, Table S6, ESM) (see also 1:1 line in Fig. 3B, C).

In addition, we applied a two-phase partitioning model to calculate the amount of PBDEs, PCBs, and BPA potentially having leached into the leachate water from e-waste based on equilibrium partitioning. Calculated aqueous concentrations ranged from 8.3E − 18 mol/L_ASW_ for hexabromobiphenyl #153 to 6.5E − 07 mol/L_ASW_ for BPA (Table S4, ESM). These concentrations were used to calculate relative effect contributions of each individual chemical (*i*) (EU_chem (*i*)_) and summed up in a concentration-additive mixture model (EU_chem_). Applying this so-called iceberg modelling (see details in section S4 Iceberg Modelling, Table S3, Table S4, ESM), we could explain less than 1.5% of the observed biological effects of the parameters fluorescence and cell density by EU_chem_ for the EW samples (Table S7, ESM), indicating their small contribution to the overall effect.

## Discussion

### Effects from the leachates

Mostly EW and KB resulted in measurable adverse effects on the microalgae test system. A reason for the strong toxicity caused by their leachates may be the high amount of additives that has been determined in the EW and can be assumed to be present in the KB. Previous chemical-analytical studies revealed high concentrations of PBDEs, PCBs, and BPA (e.g., ∑BDE-10 up to 210 mg/kg_EW_, ∑PCB_7_ up to 1.3 mg/kg_EW_, and BPA up to 188 mg/kg_EW_) in the EW material [14–16]. The KB, as an electronic device, was assumed to contain substantial amounts of additives for its application purpose. Although these substances may not act specifically on the photosystem, as indicated by their low potency to inhibit the photosynthetic capacity, their relatively high inhibition potency on the growth-based parameters (Figs. 1 and 2) indicates their environmental relevance. Baseline toxicity is the minimum toxicity a chemical can exhibit which is concentration-additive, and thereby a diverse range of different chemicals in EW and KB may act jointly in baseline toxicity.

Estimating the effect contribution of the sum of chemicals previously measured in the exhaustive solvent-extracted EW (PBDEs, PCBs, and BPA) [14–16] and that can be expected to be present in the leachates, we could explain < 1.5% of the observed biological effects (see Table S7, ESM). Although this modeled data is based on several assumptions such as equilibrium partitioning and QSAR-derived effect concentrations, we can draw two conclusions: firstly, despite advanced instrumentation and in-depth chemical analyses by exhaustive solvent extraction and targeted gas chromatography coupled to mass spectrometry [14–16], there is still a substantial lack of knowledge regarding which representatives of a wide universe of chemicals present in EW contribute to the observed effects. Secondly, even though the modeled data presented above was based on a worst case scenario assuming equilibrium partitioning conditions which is unlikely in the applied relatively short-term 4-day weathering experiment, the explained effects remained in a very low range (> 98% of the effects remained unexplained by the model). Comparably low fractions of toxicity explained by advanced chemical analytical tools were found in other studies (e.g., Neale et al. [31]). This fact highlights the great benefit of effect-based tools for risk assessment of potential hazards from plastic leachates, which could hardly be predicted by chemical analytical tools, even if advanced methods were applied, as, e.g., in the studies of Arp et al. [14] and Morin et al. [15, 16]. Capolupo and colleagues [32] directly linked the high toxicity of leachates of car tire rubber and polyvinyl chloride (PVC) on freshwater and marine microalgae to high contents of additives. A study by Chae et al. [33] investigated the toxicity of expanded polystyrene (EPS) towards four microalgae species. Generally, the photosynthetic activity of all four species was enhanced by EPS leachates [33]. The authors hypothesized about this hormesis effect that leaching DOC (such as the measured hexabromocyclododecanes, BPA, and UV-absorber UV326) might have promoted photosynthetic activity and thereby cell growth [33]. Previous studies have also explained such observations by trace concentrations of plastic additive chemicals [34].

Leachates from additive-free pre-production polymers PET, PP, and PS did not show detectable ecotoxicological effects on algae growth or photosynthetic activity in our study (Fig. 2, Table S5, ESM). The measurable effects barely differed from the respective blanks as seen for the parameter cell density (grey shaded area, Fig. 2B) indicating low risks from the virgin material of these polymers itself. This means that no specific toxicity such as inhibition of the photosystem was induced by substances leaching from weathered PET, PP, and PS microplastic in UV light or in dark treatments. One reason for the relatively low ecotoxicological potential of pre-production plastics may be the absence of additives and toxic degradation products of PET, PP, and PS.

Whereas in our study only PE showed algae toxicity to some extent, Tetu et al. [10] detected impaired growth, photosynthetic capacity, and genome-wide transcriptional changes by PE and PVC leachates for an important primary producer, *Prochlorochoccus spec*. Whereas adverse effects of plastic leachates were reduced by weathering in one study [35], we identified 2- to threefold increased toxicity for PE_UV_ compared to PE_DC_. Interestingly, KB showed similar reduced effects upon weathering as described by Tetu et al. [10] which could be indicative for the photo-degradation of toxic substances leaching from the material, to less toxic transformation products. Similar to the results of Rummel et al. [17], prominent effects were caused by EW with EC_50_ values of REFs around or below EC_50_ ≤ 1 (REF) (or reciprocal EU values ≥ 1 (1/REF)) meaning that no dilution or enrichment from the original 200 mL leachate was necessary to target the observed effect. Contrarily, the leachates from pre-production polymers had to be enriched by factors of 18 to 190 to elicit effects.

### MoA of PE degradation products

The observed elevated EU values for PE_UV_ compared to PE_DC_ may potentially be the result of photo-oxidizing of the PE polymer, resulting in larger fractions of polymer breakdown products in the leachates (Fig. 2, Table S5, ESM). Gewert et al. [20] and Rummel et al. [17] identified degradation products in leachates that were generated using the identical weathering setup. Similar to Rummel et al. [17], where monocarboxylic acids showed more explicit effects in cells than dicarboxylic acids of comparable carbon chain length, the monocarboxylic acids showed a higher potential to elicit adverse effects on microalgae. The analytically identified degradation products of PE, mainly dicarboxylic acids of different chain lengths (C5–C18), showed effects on all measured parameters; however, only acids with short carbon chain length (< C10) and at high tested concentration were active (Table 1). Still a trend of increasing effects with increasing carbon chain length could be observed. This increase may relate to the acids’ linear relationship between membrane permeability, as can be correlated with the hexadecane/water partition coefficient [36].

Applying the modified QSAR of Altenburger et al. [21], we predicted baseline toxicity for the investigated carboxylic acids and calculated resulting TRs. The observed TRs were < 10 for almost all acids for the effect parameters fluorescence and cell density and were in good agreement with the non-specific disturbance of the cell membrane elicited by the acids. Moreover, the growth-based effect parameters of the microalgae test displayed a very narrow range of TRs indicating good agreement between calculated and measured EC_50_ values supporting their baseline toxic MoA (Fig. 3A). Based on the values of 1 < TR < 10 for the effect parameters fluorescence and cell densities (Fig. 3A), it can be assumed that the critical membrane concentration of 70 mmol/L_lip_ resulting in destabilization of the phospholipid bilayer was reached by the carboxylic acids [37], but there were no specific effects on the photosystem II. The applied QSAR may potentially not be adequate for photosystem II inhibition since its main purpose was to calculate baseline toxicity values for integrated parameters like growth inhibition.

Cellular membranes contain unsaturated fatty acids [38] that are especially prone to attack by free radicals causing lipid peroxidation [39], lysis [40], and fatty acid deesterification [41]. In this context, fluorescence as a measure of growth is a good indicator of membrane disintegration, which is dependent on the hydrophobicity of the exposed chemicals. Fluorescence could therefore indicate baseline toxicity. These observations compare well to the finding discussed above that the apical parameters fluorescence and cell densities were the most sensitive parameters in the leachate tests (Fig. 1). Furthermore, the EU values of fluorescence correlated statistically significantly with the cytotoxicity values derived from reporter gene assays with regression slopes close to one (Fig. 3B, C, Table S6, ESM). The similarly good correlation to cytotoxicity of reporter gene assays therefore suggests that the impairment of the photosystem is an indirect effect of baseline toxicity (Fig. 3B, C, Table S6, ESM).

In a comparable way, the AREc32 cell assay, responsive to oxidative stress, was induced across all tested polymer types in Rummel et al. [17]. At high physiological concentrations, reactive oxygen species may cause cell damage and cell death [42] often induced by small reactive molecules [43]. Another indication for baseline toxicity as the underlying mechanism of toxicity was the good correlation and regression slopes close to 1 between cytotoxicity values (TU_bio_) from the AhR assay and EU_bio_ values of the AREc32 assay (Fig. 3, Table S6, ESM). Accuracy of the linear regression models could potentially be improved in the experimental setup if the same amount of plastic was used for the leaching experiment. In Rummel et al. [17], 50 g of each plastic type was leached into artificial seawater whereas in this study 40 g were applied in the weathering setup (note that this slight experimental difference is not reflected in the given concentrations in the assay, since identical enrichment factors, based on the same underlying volumetric measure of 200 mL ASW in the weathering setup, were applied).

## Conclusion

 Additive-containing EW and KB caused strong adverse effects on microalgae growth and photosynthesis while leachates from virgin pre-production polymers PET, PP, and PS elicited effects in the range of the blanks. Though several target pollutants were identified in the EW, the known, quantified ones could only account for a small fraction of the observed effects (< 1.5%), indicating that other pollutants drive the observed toxicity, assuming the modeled assumptions were accurate. Elevated toxicity by UV-treated PE_UV_ leachates could potentially be explained by the presence of small reactive molecules such as mono- and dicarboxylic acids that were very likely present in the leachates because of photo-oxidation. These degradation products were mainly baseline toxic since the measured data were consistent with predicted baseline toxicity values for the investigated carboxylic acids. Our findings highlight that degrading and largely additive-free pre-production polymers have the potential to induce adverse effects on whole organisms, particularly PE; however, stronger adverse effects were observed if the polymers contained additives or other chemicals. As a future step, advanced chemical analytical tools, e.g., effect-directed analysis combined with non-target screening, would be necessary to resolve the chemicals causing these effects [44, 45]. Therefore, the application of effect-based tools provides a reliable strategy to assess potential environmental risks from potentially hazardous materials like plastics. To increase our understanding of chemical leachate vs. particle toxicity of plastics, future studies should investigate algae toxicity of migrating additives and compare them to toxicity caused by the mere presence of the particles, benchmarked to natural particle concentrations.

## Supplementary Information

Below is the link to the electronic supplementary material.

Supplementary file1 (PDF 1141 kb)
