# Supplementary material for: Effects of leachates from UV-weathered microplastic on the microalgae Scenedesmus vacuolatus
Source: Anal Bioanal Chem. 2021 Dec 22;414(4):1469–79. doi: 10.1007/s00216-021-03798-3 (PMC8761717; doi:10.1007/s00216-021-03798-3)
Supplement: Supplementary file 1 — Supplementary file1 (PDF 1141 kb) [file 216_2021_3798_MOESM1_ESM.pdf]

Electronic Supplementary Material (ESM)

Effects of leachates from UV-weathered microplastic on the microalgae *Scenedesmus vacuolatus*

Christoph Rummel, Hannah Schäfer, Annika Jahnke, Hans Peter H. Arp and Mechthild Schmitt-Jansen*

*corresponding author's Email: mechthild.schmitt@ufz.de

Table of content

Section S1 Test Polymers. 2

Section S2 Data Analyses 4

Section S3 QSAR Data 6

Section S4 Iceberg Modelling 7

References 14

List of Tables

Table S1: Summary of mono- and dicarboxylic acids tested in the microalgae assays 4

Table S2: Physico-chemical properties of mono- and dicarboxylic acids 6

Table S3: Chemical data used for modelling the chemical partitioning 9

Table S4: Results of the partitioning model, the QSAR and the iceberg modelling 10

Table S5: Effect units (EU) of the microalgae assays 11

Table S6: Parameters of the linear models. 13

Table S 7: Results of the iceberg modelling 13

List of Figures

Figure S 1: Artificial weathering setup 3

Figure S 2: The investigated microalgae *Scenedesmus vacuolatus* 5

Section S1 Test Polymers.

The test polymers PE, PET, PS and PP were purchased from Goodfellow (Hamburg, Germany) as raw resin pellets containing no additives (supplier information). The selection of the polymer types was done based on polymer's availability as additive-free resins and their relevance for the European market. The selected polymers belong to the top seven polymers based on European demand of 2-10 M t in 2018 [1]. A detailed description of the experimental setup of the weathering treatment can be found in the supporting information (SI) in Rummel et al. [2]. In short, the raw resin pellets were milled to a size range below 350 µm by the company Messer (Messer Group GmbH, Bad Soden, Germany). E-waste and keyboard granules were cryomilled to similar size ranges at the laboratory. Instant Ocean® (Blacksburg, Virginia, USA) was used to prepare the artificial seawater at 35 g/L. 40 g of each polymer type was exposed to UV-irradiation by an OSRAM Supratec HTC400-241 R7s UVA/UVB lamp (main UV A and B in the range of 314 – 400 nm) for four days. This artificial weathering simulated around 410 days of middle European outdoor exposure (see SI in Rummel et al., 2019). After the leaching period, microplastic leachates were filtered on a 40 µm stainless steel mesh to remove the particles and the chemicals present in the leachates were concentrated via solid-phase extraction (see SPE protocol SI Rummel et al., 2019).

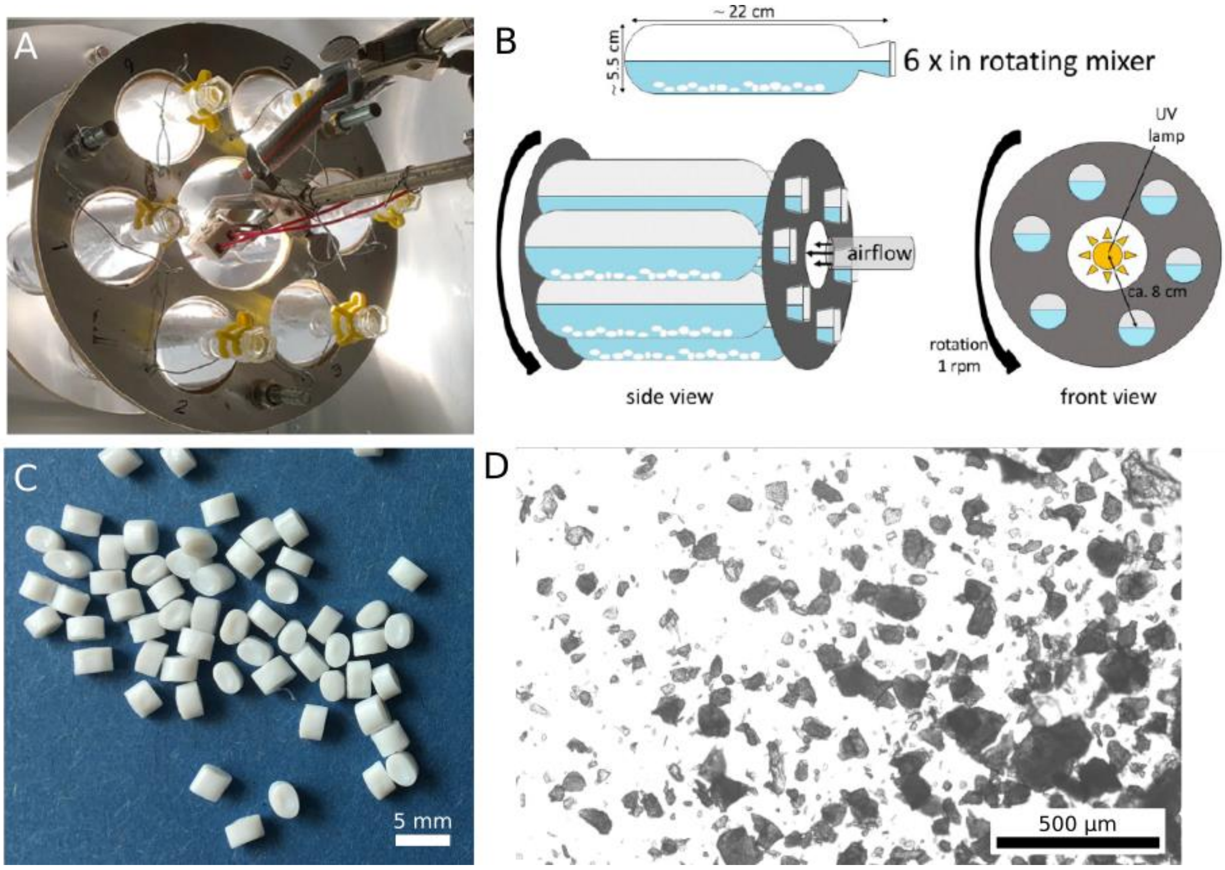

Figure S 1: Artificial weathering setup (A & B) and size ranges of the investigated polymer material (here: PET). Source panel **A&B**: Gewert et al. [3], **https://pubs.acs.org/doi/full/10.1021/acs.estlett.8b00119**, with permission to reproduce the content. Further permissions related to the material excerpted should be directed to the ACS. Source panel **C&D**: Rummel et al. [2] **https://pubs.acs.org/doi/10.1021/acs.est.9b02400** with permission to reproduce the content. Further permissions related to the material excerpted should be directed to the ACS

Table S1: Summary of mono- and dicarboxylic acids tested in the microalgae assays

	IUPAC	CAS	purity (%)
**Monocarboxylic acids**	Pentanoic acid	109-52-4	≥ 99
	Heptanoic acid	111-14-8	≥ 99
	Octanoic acid	124-07-2	≥ 98
	Nonanoic acid	112-05-0	≥ 96
	Decanoic acid	334-48-5	≥ 98
	Undecanoic acid	112-37-8	≥ 99
	Dodecanoic acid	143-07-7	≥ 98
	Tetradecansäure	544-63-8	≥ 99
	Hexadecanoic acid	57-10-3	≥ 99
	Octadecanoic acid	57-11-4	≥ 98.5
**Dicarboxylic acids**	Pentanedioic acid	110-94-1	≥ 99
	Heptanedioic acid	111-16-0	≥ 98
	Octandioic acid	505-48-6	≥ 98
	Nonanedioic acid	123-99-9	≥ 98
	Decanedioic acid	111-20-6	≥ 99
	Undecanedioic acid	1852-04-6	≥ 97
	Dodecanedioic acid	693-23-2	≥ 99
	Tetradecanedioic acid	821-38-5	≥ 99

Section S2 Data Analyses

For the calculation of the relative inhibition of microalgae growth (in percent (%)) (see image of synchronized culture of S. vacuolatus **Figure S2 A**) the Chl *a* autofluorescence was compared to the mean value of the controls (eq. S1). The For the other endpoints (cell density, yield I and II after 2 h and 24 h after dosing), the relative inhibition (in percent (%)) without background subtraction was calculated (eq. S2).

$$Growth\ rate = \frac{autofluorescence_{\,24\,\mathrm{h}} - autofluorescence_{\,2\,\mathrm{h}}}{autofluorescence_{\,24\,\mathrm{h}}} \qquad \text{(eq. S1)}$$

$$Inhibition\ [\%] = \left(1 - \frac{endpoint\ value_{\,\mathrm{sample}}}{mean\ endpoint\ value_{\,\mathrm{control}}}\right) * 100 \qquad \text{(eq. S2)}$$

Endpoint $value_{sample}$ represents the measured cell density or the photosynthetic YI and YII after 2 h or 24 h. An example for the determination of cell density using FACSCelesta is given in **Figure S2 B and C**. The mean endpoint $value_{control}$ comprises the averaged measured values for the respective endpoints for the unexposed negative control cells. Dose-response curves and EC_{50}

values were calculated for the derived endpoints applying a 4-parametric Hill model (between 0 % and 100 % and with slope and EC_{50} as adjustable parameters) using the software SigmaPlot 13.0.

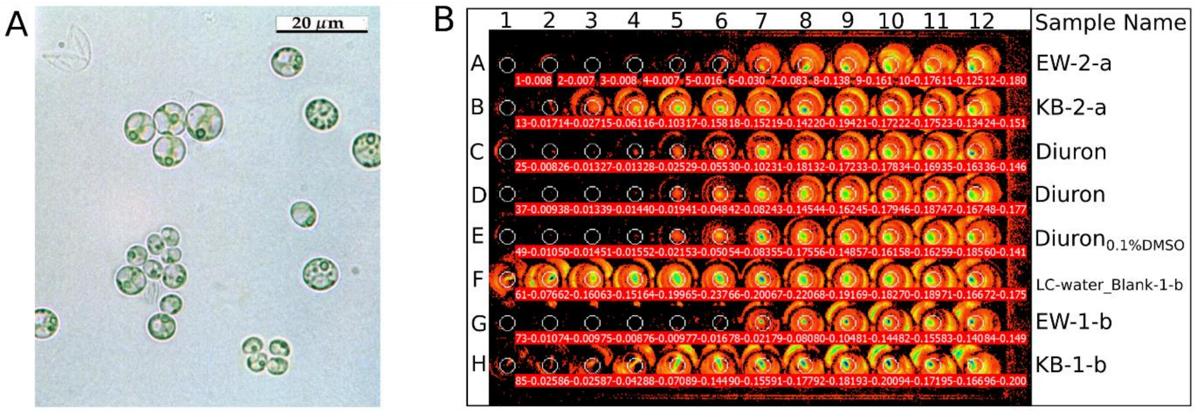

Figure S 2: The investigated microalgae *Scenedesmus vacuolatus* in the growth inhibition test using Imaging PAM. **A:** Miscroscopy image of the synchronized culture. Scale bar represents 20 µm. **B:** Exemplified results of the Imaging PAM measurements for the maximal fluorescent yield, *Fm*, of different samples (EW and KB, reference compound Diuron and a LC-water Blank) as rows (A-H) in a dilution series (1-12). Corresponding sample names are presented right hand side next to the plate. Color coding corresponds to the *Fm*

Section S3 QSAR Data

Table S2: Physico-chemical properties of mono- and dicarboxylic acids and calculated speciation. Details on the calculation of logD_{lipw} [pH7.0] see eq. 1-3 in the main manuscript

substance	pKa at 25°C	Reference accessed on 12th of April 2018	fraction$_{neutral}$ (f_{HA})	fraction$_{ionized}$ (f_{A-} or 1-f_{HA})	logD_{lipw} [L_w/L_{lip} or L_w/kg_{lip}] [4]
pentanoic acid	4.8	pubchem: DEAN.JA (1987)	6.27E-03	9.94E-01	0.32
pentandioic acid	4.3	pubchem: KORTUM.G ET AL (1961)	1.99E-03	9.98E-01	-1.42
heptanoic acid	4.9	pubchem: DEAN.JA (1987)	7.88E-03	9.92E-01	1.33
heptandioic acid	4.5	pubchem: KORTUM.G ET AL (1961)	3.15E-03	9.97E-01	-0.50
octanoic acid	4.9	pubchem: DEAN.JA (1987)	7.88E-03	9.92E-01	2.04
octandioic acid	assumed 4.9		7.88E-03	9.92E-01	-0.08
nonanoic acid	5	pubchem: DEAN.JA (1987)	9.90E-03	9.90E-01	2.35
nonandioic acid	4.6	pubchem: KORTUM.G ET AL (1961)	3.97E-03	9.96E-01	0.51
decanoic acid	4.9	pubchem: BARRATT.MD (1996)	7.88E-03	9.92E-01	3.05
decandioic acid	assumed 4.9		7.88E-03	9.92E-01	0.63
undecanoic acid	assumed 4.9		7.88E-03	9.92E-01	3.35
undecandioic acid	assumed 4.9		7.88E-03	9.92E-01	1.33
dodecanoic acid	5.3	pubchem: SERJEANT.EP & DEMPSEY.B (1979)	1.96E-02	9.80E-01	3.60
dodecandioic acid	assumed 4.9		7.88E-03	9.92E-01	1.74
tetradecanoic acid	4.9	pubchem: BARRATT.MD (1996)	7.88E-03	9.92E-01	5.07
tetradecandioic acid	assumed 4.9		7.88E-03	9.92E-01	1.94
hexadecanoic acid	4.8	predicted: SPARC; 2008	6.27E-03	9.94E-01	6.18
octadecanoic acid	4.8	predicted: SPARC; 2008	6.27E-03	9.94E-01	7.19

Section S4 Iceberg Modelling

To model the potential effect contribution of each individual chemical (chem(*i*)) in a so-called iceberg modelling approach, a few assumptions and simplifications were made. Firstly, we needed to estimate the potential concentration of each chemical (*i*) in the leachate. To estimate chemical partitioning in a two-phase equilibrium model the dry mass concentrations in the applied EW sample were used. Mass concentrations of brominated diphenyl ethers (PBDEs), polychlorinated biphenyls (PCBs) and bisphenol A (BPA) of this specific EW were taken from published literature [5-7]. Assuming the worst case scenario of chemical equilibrium in the applied setup of four days of leaching, the mass concentrations in the leachate water were calculated based on eq. S3 – S6.

$$f_{i\mathrm{EW}} = \frac{1}{1+\frac{V_{\mathrm{ASW}}}{K_{\mathrm{iEW/water}} * m_{\mathrm{EW}}}} \quad \text{(eq.S3)}$$

$$f_{i\mathrm{ASW}} = \frac{1}{1+K_{i\mathrm{EW/water}} * \frac{m_{\mathrm{EW}}}{V_{\mathrm{ASW}}}} \quad \text{(eq.S4)}$$

$$c_{i\mathrm{EW}} = \frac{f_{i\mathrm{EW}} * m_{\mathrm{itot}}}{m_{\mathrm{EW}}} \quad \text{(eq.S5)}$$

$$c_{i\mathrm{ASW}} = \frac{f_{\mathrm{iASW}} * m_{\mathrm{itot}}}{V_{\mathrm{ASW}}} \quad \text{(eq.S6)}$$

$f_{i\mathrm{EW}}$= fraction (*i*) in plastic (EW)

$f_{i\mathrm{ASW}}$ = fraction (*i*) in ASW

m_{EW}= mass of plastic (EW)

V_{ASW}= Volume of ASW

$K_{i\mathrm{EW/ASW}}$= partition constant of (*i*) between plastic (EW) and ASW (here $K_{i\,\mathrm{ow}}$ [L/kg])

$c_{i\mathrm{EW}}$ = mass concentration (*i*) in plastic (EW)

$c_{i\mathrm{ASW}}$ = mass concentration (*i*) in ASW

$m_{i\mathrm{tot}}$= total mass of compound (*i*)

Secondly, the logK_{ow} values for each chemical BDEs 28, 47, 99, 100, 153, 154, PCBs 52, #101, #118, #138, #153, #180, Pentabromoethylbenzene (PBEB), Hexabromobenzene (HBB), HBB 153 and BPA was calculated using the UFZ-LSER database applying existing Linear Solvation Energy Relationship (LSER) parameters [8]. We assumed the log$K_{\mathrm{polymer\text{-}water}}$ (unknown polymer composition of EW) partition coefficient to be in good agreement with the log K_{ow} [9,10] and therefore used the logK_{ow} as an approximation. The logK_{ow}-based QSAR by Altenburger et al. [11] was applied to estimate the chemicals EC_{50} concentration (see eq. 1 main manuscript). Then, effect units derived from the chemical partitioning model ($EU_{\mathrm{chem}(i)}$) were calculated as the ratio of the

predicted concentration c_i of a chemical i and its predicted EC_{50} value (eq. S7). Assuming concentration addition in the mixture model, the sum of $EU_{chem(i)}$ resulting in EU_{chem} (eq. S8) was calculated to roughly explain the percent observed biological effects in the microalgae assay by a certain contribution of each individual chemical i (eq. S9). The results of this modelling approach are presented in Table S2.

$$EU_{chem\,(i)} = \frac{c_i}{EC_{50(i)}} \qquad (eq.\,S7)$$

$$EU_{chem} = \sum_{i=1}^{n} EU_{chem\,(i)} = \sum_{i=1}^{n} \frac{c_i}{EC_{50\,(i)}} \qquad (eq.\,S8)$$

$$\text{Effect explained [\%]} = \frac{EU_{chem}}{EU_{bio\ (fluo\ or\ cell)}} * 100 \qquad (eq.\,S9)$$

Table S3: Chemical data used for modelling the chemical partitioning of chemical (*i*) in a two-phase system

**Chemical (*i*)**	**CAS**	**mol mass**	**mass concentration chemical (*i*) per EW dry weight [ng_i/kg_{EW}]** [5-7]	**total mass of chemical (*i*) in two-phase system (ng) $m_{i\,tot}$**	**log K_{ow} (LSER)[L_{water}/L_{oct}]**
Tri-BDE 28	41318-75-6	406.90	88.4	4.42E+00	6.22
Tetra-BDE 47	5436-43-1	485.79	835.9	4.18E+01	6.73
Penta-BDE 99	60348-60-9	564.69	27991.8	1.40E+03	8.29
Penta-BDE 100	189084-64-8	564.69	5303.5	2.65E+02	7.22
Hexa-BDE 153	68631-49-2	643.58	1071982.2	5.36E+04	7.68
Hexa-BDE 154	207122-15-4	643.58	312570.8	1.56E+04	7.67
Hepta-BDE 183	207122-16-5	722.48	4471828.8	2.24E+05	8.19
PBEB	85-22-3	500.65	707.0	3.53E+01	7.17
HBB	87-82-1	551.49	889.3	4.45E+01	6.91
Hexa-BB 153	59080-40-9	627.58	0.2	1.16E-02	7.65
Tetra-PCB 52	35693-99-3	257.54	7064	3.53E+02	5.42
Penta-PCB 101	37680-73-2	291.99	7293	3.65E+02	5.61
Penta-PCB 118	31508-00-6	326.43	4108	2.05E+02	6.1
Hexa-PCB 153	35065-27-1	360.88	2472	1.24E+02	6.43
Hexa-PCB138	35065-28-2	360.88	2568	1.28E+02	6.59
Hepta-PCB 180	35065-29-3	395.32	408	2.04E+01	6.67
BPA	80-05-7	228.29	47852	2.39E+03	2.5

Table S4: Results of the partitioning model, the QSAR and the iceberg modelling resulting in effect units$_{chem}$ ($EU_{chem\ (i)}$) of each individual compound *i* and in the summed EU_{chem}, assuming concentration addition. (see details in **Section S4 Iceberg Modelling)**

**Chemical (*i*)**	**fraction (*i*) in EW ($f_{i\,EW}$)**	**fraction (*i*) in ASW ($f_{i\,ASW}$)**	**mass conc. (c_i) in EW [ng_i/kg_{EW}]**	**mass conc. (c_i) in ASW (g_i/L_{ASW})**	**mass conc. (*i*) in ASW (mol/L_{ASW})**	**Altenburger QSAR (EC_{50} [mol/L])**	**$EU_{chem(i)}$** (eq. S7)	**$\sum EU_{chem(i)}$ = EU_{chem}** (eq.S8)
Tri-BDE 28	0.999	2.4E-06	8.8E+01	5.3E-11	1.3E-13	5.4E-07	2.4E-07	**0.006**
Tetra-BDE 47	0.999	7.4E-07	8.4E+02	1.6E-10	3.2E-13	2.0E-07	1.6E-06	
Penta-BDE 99	0.999	2.1E-08	2.8E+04	1.4E-10	2.5E-13	8.9E-09	2.9E-05	
Penta-BDE 100	0.999	2.4E-07	5.3E+03	3.2E-10	5.7E-13	7.4E-08	7.6E-06	
Hexa-BDE 153	0.999	8.4E-08	1.1E+06	2.2E-08	3.5E-11	3.0E-08	1.2E-03	
Hexa-BDE 154	0.999	8.6E-08	3.1E+05	6.7E-09	1.0E-11	3.0E-08	3.4E-04	
Hepta-BDE 183	0.999	2.6E-08	4.5E+06	2.9E-08	4.0E-11	1.1E-08	3.7E-03	
Pentabromoethylbenzene (PBEB)	0.999	2.7E-07	7.1E+02	4.8E-11	9.5E-14	8.2E-08	1.2E-06	
Hexabromobiphenyl (HBB)	0.999	4.9E-07	8.9E+02	1.1E-10	2.0E-13	1.4E-07	1.4E-06	
Hexa-BB 153	0.999	9.0E-08	2.3E-01	5.2E-15	8.3E-18	3.2E-08	2.6E-10	
Tetra-PCB 52	0.999	1.5E-05	7.1E+03	2.7E-08	1.0E-10	2.7E-06	3.9E-05	
Penta-PCB 101	0.999	9.8E-06	7.3E+03	1.8E-08	6.1E-11	1.8E-06	3.4E-05	
Penta-PCB 118	0.999	3.2E-06	4.1E+03	3.3E-09	1.0E-11	6.9E-07	1.4E-05	
Hexa-PCB 153	0.999	1.5E-06	2.5E+03	9.2E-10	2.5E-12	3.6E-07	7.1E-06	
Hexa-PCB 138	0.999	1.0E-06	2.6E+03	6.6E-10	1.8E-12	2.6E-07	7.0E-06	
Hepta-PCB 180	0.999	8.6E-07	4.1E+02	8.7E-11	2.2E-13	2.2E-07	9.9E-07	
BPA	0.987	1.2E-02	4.7E+04	1.5E-04	6.5E-07	8.8E-04	7.4E-04	

Table S5: Effect units (EU) as the inverse EC_{50} values of the microalgae endpoints fluorescence (fluo), cell density (cell), and yield I and II (YI, II) after 2h and 24h after dosing with respective standard error (SE). Values are in the units of 1/REF (relative enrichment factor).

Sample name	Replicate #	EU_fluo	SE EU_{fluo}	EU_{cell}	SE EU_{cell}	EU_{YI2h}	SE EU_{YI2h}	EU_{YII2h}	SE EU_{YII2h}	EU_{YI24h}	SE EU_{YI24h}	EU_{YII24h}	SE EU_{YII24h}
SPE blank	1	ND	ND	ND	ND	ND	ND	ND	ND	ND	ND	ND	ND
	2	ND	ND	5.8E-03	1.5E-03	ND	ND	8.1E-03	2.2E-03	ND	ND	ND	ND
	3	ND	ND	ND	ND	ND	ND	ND	ND	ND	ND	ND	ND
	4	ND	ND	ND	ND	ND	ND	ND	ND	ND	ND	ND	ND
proc.blank_DC	1	6.2E-03	2.7E-03	5.1E-03	9.5E-03	ND	ND	1.1E-02	1.1E-02	5.1E-03	3.6E-01	6.0E-03	5.5E-04
	2	ND	ND	8.6E-03	1.6E-03	ND	ND	ND	ND	ND	ND	ND	ND
	3	ND	ND	1.3E-02	9.4E-04	ND	ND	ND	ND	ND	ND	ND	ND
proc.blank_UV	1	ND	ND	2.6E-02	3.4E-02	ND	ND	ND	ND	5.7E-03	5.7E-04	7.0E-03	8.4E-04
	2	ND	ND	8.6E-03	1.4E-03	ND	ND	ND	ND	ND	ND	ND	ND
	3	ND	ND	1.6E-02	1.8E-03	ND	ND	ND	ND	ND	ND	ND	ND
EW_DC	1	9.4E-01	1.1E-01	9.0E-01	1.4E-01	6.4E-01	1.1E-01	1.1E+00	7.3E-01	1.7E-01	5.6E-02	2.2E-01	7.6E-02
	2	6.8E-01	4.6E+00	9.6E-01	1.3E-01	4.3E-01	1.3E-01	2.8E-01	7.6E-02	4.2E-01	1.7E-01	2.3E-01	8.7E-02
	3	1.0E+00	1.6E-01	6.3E-01	1.0E-01	5.0E-01	1.4E-01	5.1E-01	2.2E-01	2.7E-01	8.2E-02	4.4E-01	7.1E-02
EW_UV	1	4.9E-01	1.0E-01	1.3E+00	1.5E+00	5.5E-01	3.6E-01	6.1E-01	3.5E-01	1.7E-01	1.8E-02	2.8E-01	5.2E-02
	2	1.1E+00	1.7E-01	7.8E-01	5.2E-02	7.2E-01	2.3E-01	3.9E-01	1.2E-01	5.5E-01	2.3E-01	2.9E-01	3.7E-02
	3	9.3E-01	2.7E-01	7.2E-01	1.4E-01	4.1E-01	8.5E-02	7.1E-01	3.5E-01	3.0E-01	7.1E-02	4.6E-01	5.3E-02
KB_DC	1	1.1E-01	7.0E-03	1.3E-01	1.9E-02	6.7E-02	7.1E-03	3.9E-02	4.1E-03	3.5E-02	2.2E-02	3.0E-02	7.5E-03
	2	1.4E-01	2.6E-02	8.8E-02	1.9E-02	6.7E-02	1.1E-02	1.3E-01	4.3E-02	2.3E-02	6.9E-03	3.5E-02	5.3E-03
	3	1.4E-01	3.5E-02	9.2E-02	1.6E-02	7.3E-02	1.7E-02	4.8E-02	4.9E-03	1.9E-02	4.7E-03	2.5E-02	6.5E-03
KB_UV	1	9.1E-02	1.5E-02	1.7E-02	8.3E-03	2.2E-02	3.6E-03	1.8E-02	2.9E-03	1.4E-02	9.9E-04	1.3E-02	1.3E-03
	2	1.0E-01	1.9E-02	3.0E-02	9.0E-03	4.0E-02	4.6E-03	5.1E-02	9.9E-03	9.3E-03	1.8E-03	1.3E-02	3.4E-03
	3	8.1E-02	2.1E-02	3.5E-02	1.4E-02	5.1E-02	1.0E-02	4.2E-02	1.1E-02	1.4E-02	2.7E-03	1.8E-02	2.2E-03
PE_DC	1	1.5E-02	5.2E-03	1.6E-02	3.5E-03	ND	ND	1.7E-02	1.5E-02	7.9E-03	1.3E-03	1.2E-02	1.0E-03
	2	9.8E-03	2.9E-03	1.2E-02	8.8E-03	5.8E-03	2.3E-03	6.7E-03	3.1E-03	ND	ND	ND	ND
	3	6.7E-03	3.7E-03	5.8E-03	3.4E-03	ND	ND	1.6E-02	5.9E-03	ND	ND	ND	ND

**Sample name**	**Replicate #**	**EU_fluo**	**SE EU_{fluo}**	**EU_{cell}**	**SE EU_{cell}**	**EU_{YI2h}**	**SE EU_{YI2h}**	**EU_{YII2h}**	**SE EU_{YII2h}**	**EU_{YI24h}**	**SE EU_{YI24h}**	**EU_{YII24h}**	**SE EU_{YII24h}**
PE_UV	1	3.2E-02	1.0E-02	4.1E-02	1.4E-02	ND	ND	1.5E-02	4.4E-03	1.1E-02	2.0E-03	2.0E-02	3.6E-01
	2	1.9E-02	4.5E-03	3.3E-02	1.3E-02	1.5E-02	4.6E-03	3.4E-02	6.6E-03	ND	ND	ND	ND
	3	2.7E-02	6.7E-03	5.6E-02	1.9E-02	4.0E-02	1.5E-02	7.5E-02	1.5E-02	ND	ND	6.1E-03	8.6E-04
PET_DC	1	ND	ND	ND	ND	ND	ND	ND	ND	ND	ND	ND	ND
	2	ND	ND	8.3E-03	3.4E-03	ND	ND	ND	ND	ND	ND	ND	ND
	3	1.0E-02	6.4E-02	1.3E-02	2.8E-03	ND	ND	ND	ND	ND	ND	ND	ND
PET_UV	1	7.7E-03	1.8E-03	ND	ND	ND	ND	1.4E-02	7.8E-03	ND	ND	7.8E-03	3.6E-03
	2	7.3E-03	2.7E-03	1.7E-02	3.4E-03	ND	ND	ND	ND	ND	ND	ND	ND
	3	1.3E-02	2.7E-03	1.2E-02	2.6E-03	ND	ND	ND	ND	ND	ND	ND	ND
PS_DC	1	ND	ND	ND	ND	ND	ND	ND	ND	ND	ND	ND	ND
	2	ND	ND	ND	ND	ND	ND	ND	ND	ND	ND	ND	ND
	3	ND	ND	1.4E-02	1.3E-03	#DIV/0!	ND	ND	ND	ND	ND	ND	ND
PS_UV	1	1.1E-02	5.2E-03	1.4E-02	1.6E-03	ND	ND	5.4E-03	2.9E-03	5.3E-03	1.1E-03	9.6E-03	3.5E-04
	2	9.2E-03	3.2E-03	1.3E-02	2.5E-03	ND	ND	ND	ND	ND	ND	ND	ND
	3	ND	ND	1.3E-02	3.2E-03	ND	ND	ND	ND	ND	ND	ND	ND
PP_DC	1	8.3E-03	2.5E-03	ND	ND	ND	ND	1.0E-02	4.5E-03	ND	ND	ND	ND
	2	ND	ND	8.8E-03	3.4E-03	8.3E-03	3.1E-03	ND	ND	ND	ND	ND	ND
	3	5.8E-03	6.0E-04	1.5E-02	2.2E-03	ND	ND	ND	ND	ND	ND	ND	ND
PP_UV	1	1.1E-02	4.1E-03	4.2E-02	4.0E-02	7.5E-03	3.6E-03	3.9E-02	2.4E-02	7.2E-03	1.4E-03	9.9E-03	2.8E-03
	2	ND	ND	9.4E-03	4.7E-03	ND	ND	ND	ND	ND	ND	ND	ND
	3	9.3E-03	2.9E-03	2.4E-02	1.5E-03	ND	ND	ND	ND	ND	ND	5.5E-03	3.5E-03

ND = not detected at REF < 198

Table S6: Parameters of the linear models (y=*a*x +*b*) using the microalgae endpoints (EU_{fluo}, EU_{cell}, EU_{YI24h} and EU_{YII24h}) the as y-variable as a function of the cytotoxicity values (TU_{bio}) of the reporter gene results from Rummel et al. (2019) as x-variable.

y-variable	x-variable	Slope *a*	Intercept *b*	Coefficient of determination R^2	*p* - value
EU_{fluo}	TU_{bio} AhR	1.2	1.1	0.65	<0.0001
	TU_{bio} AREc32	1.6	3.5	0.38	0.003
	TU_{bio} PPARγ	-0.4	-3.3	0.0003	0.32
EU_{cell}	TU_{bio} AhR	0.8	1.2	0.08	0.05
	TU_{bio} AREc32	2.6	7.3	0.42	<0.01
	TU_{bio} PPARγ	-0.29	-2.3	-0.02	0.56
EU_{YI24h}	TU_{bio} AhR	1.5	2.2	0.83	<0.0001
	TU_{bio} AREc32	2.3	5.9	0.44	<0.05
	TU_{bio} PPARγ	-0.1	-1.7	-0.07	0.89
EU_{YII24h}	TU_{bio} AhR	1.3	1	0.88	<0.0001
	TU_{bio} AREc32	2.2	4.9	0.52	<0.01
	TU_{bio} PPARγ	-0.3	-3.3	-0.04	0.58

Table S 7: Results of the iceberg modelling which explains the percent [%] observed biological effect of the endpoint fluorescence or cell number by EU_{chem} (see details section ESM 3 iceberg modelling, Table S3, Table S4)

$\sum EU_{chem(i)}$ = EU_{chem}	Sample name	Replicate #	Percent $effect_{fluo}$ explained by EU_{chem} [%] (eq. S9)	Percent $effect_{cell}$ explained by EU_{chem} [%] (eq. S9)
**6,08E-03**		1	0.64	0.67
	EW_{DC}	2	0.89	0.63
		3	0.61	0.96
		1	1.24	0.47
	EW_{UV}	2	0.54	0.78
		3	0.65	0.84

References

1. PlasticsEurope (2019) Plastic - the Facts 2019: An analysis of European plastics production, demand and waste data. Brussels, Belgium
2. Rummel CD, Escher BI, Sandblom O, Plassmann MM, Arp HPH, MacLeod M, Jahnke A (2019) Effects of Leachates from UV-Weathered Microplastic in Cell-Based Bioassays. Environ Sci Technol 53 (15):9214-9223. doi:10.1021/acs.est.9b02400
3. Gewert B, Plassmann M, Sandblom O, MacLeod M (2018) Identification of Chain Scission Products Released to Water by Plastic Exposed to Ultraviolet Light. Environmental Science & Technology Letters 5 (5):272-276. doi:10.1021/acs.estlett.8b00119
4. Endo S, Escher BI, Goss K-U (2011) Capacities of Membrane Lipids to Accumulate Neutral Organic Chemicals. Environ Sci Technol 45 (14):5912-5921. doi:10.1021/es200855w
5. Morin N, Arp HPH, Hale SE (2015) Bisphenol A in Solid Waste Materials, Leachate Water, and Air Particles from Norwegian Waste-Handling Facilities: Presence and Partitioning Behavior. Environ Sci Technol 49 (13):7675-7683. doi:10.1021/acs.est.5b01307
6. Morin NAO, Andersson PL, Hale SE, Arp HPH (2017) The presence and partitioning behavior of flame retardants in waste, leachate, and air particles from Norwegian waste-handling facilities. J Environ Sci (China) 62:115-132. doi:10.1016/j.jes.2017.09.005
7. Arp HPH, Morin NAO, Andersson PL, Hale SE, Wania F, Breivik K, Breedveld GD (2020) The presence, emission and partitioning behavior of polychlorinated biphenyls in waste, leachate and aerosols from Norwegian waste-handling facilities. Sci Total Environ 715:136824. doi:10.1016/j.scitotenv.2020.136824
8. Ulrich N, Endo S, Brown TN, Watanabe N, Bronner G, Abraham MH, Goss KU (2017) UFZ-LSER database v 3.2 [Internet].
9. Lohmann R (2012) Critical Review of Low-Density Polyethylene's Partitioning and Diffusion Coefficients for Trace Organic Contaminants and Implications for Its Use As a Passive Sampler. Environ Sci Technol 46 (2):606-618. doi:10.1021/es202702y
10. Endo S, Hale SE, Goss K-U, Arp HPH (2011) Equilibrium Partition Coefficients of Diverse Polar and Nonpolar Organic Compounds to Polyoxymethylene (POM) Passive Sampling Devices. Environ Sci Technol 45 (23):10124-10132. doi:10.1021/es202894k
11. Altenburger R, Walter H, Grote M (2004) What Contributes to the Combined Effect of a Complex Mixture? Environ Sci Technol 38 (23):6353-6362. doi:10.1021/es049528k
